# Exocrine Pancreatic Maturation in Pre-term and Term Piglets Supplemented With Bovine Colostrum

**DOI:** 10.3389/fnut.2021.687056

**Published:** 2021-06-24

**Authors:** Ester Arévalo Sureda, Kateryna Pierzynowska, Björn Weström, Per Torp Sangild, Thomas Thymann

**Affiliations:** ^1^Precision Livestock and Nutrition/TERRA Teaching and Research Centre, Gembloux Agro-Biotech, University of Liège, Gembloux, Belgium; ^2^Functional Zoology, Department of Biology, Lund University, Lund, Sweden; ^3^Section of Comparative Pediatrics and Nutrition, Department of Veterinary and Animal Science, University of Copenhagen, Copenhagen, Denmark; ^4^Department of Animal Physiology, The Kielanowski Institute of Animal Nutrition and Physiology, Jabłonna, Poland; ^5^Department of Neonatology, Rigshospitalet, Copenhagen, Denmark; ^6^Department of Pediatrics, Odense University Hospital, Odense, Denmark

**Keywords:** enzymes, trypsin, amylase, lipase, immaturity, development, digestion

## Abstract

Pre-term infants have an immature digestive system predisposing to short- and long-term complications including feeding intolerance, maldigestion and necrotizing enterocolitis (NEC). Optimal feeding strategies are required to promote maturation of the gut including the exocrine pancreas. Little is known about age- and diet-related development of pancreatic exocrine enzymes following pre-term birth. Currently, bovine colostrum supplementation is investigated in clinical trials on pre-term infants. Using pigs as models for infants, we hypothesized that pancreatic enzyme content is (1) immature following pre-term birth, (2) stimulated by early colostrum supplementation, and (3) stimulated by later colostrum fortification. Thus, using piglets as models for infants, we measured trypsin, amylase, lipase and total protein in pancreatic tissue collected from piglets delivered by cesarean section either pre-term (90% gestation) or close to term. Experiment 1:Pre-term and term pigs were compared at birth and 11 days. Experiment 2: Pre-term and term pigs were either enterally supplemented with bovine colostrum or fed total parenteral nutrition for 5 days, followed by exclusive milk feeding until day 26. Experiment 3: Pre-term pigs were fed bovine's milk with or without colostrum fortification until 19 days. The results showed that pancreatic trypsin, amylase and total protein contents were reduced in pre-term vs. term pigs. Trypsin mainly increased with advancing post-conceptional age (2-fold), while amylase was affected predominantly by advancing post-natal age, and mostly in pre-term pigs from birth to 11 or 26 days. Colostrum feeding in both term and pre-term piglets decreased trypsin and increased amylase contents. Lipase activity decreased with advancing gestational age at birth and post-natal age, with no consistent responses to colostrum feeding, with lipase activities decreasing relative to total pancreatic protein content. In summary, key pancreatic enzymes, amylase and trypsin, are immature following pre-term birth, potentially contributing to reduced digestive capacity in pre-term neonates. Rapid post-natal increases occurs within few weeks of pre-term birth, partly stimulated by enteral colostrum intake, reflecting a marked adaptation capacity. Alternatively, lipase is less affected by pre-/post-natal age and feeding. Thus, there is a highly enzyme-specific and asymmetric perinatal development of the exocrine pancreas.

## Introduction

Pre-maturity, which accounts for ~10% of all live births, is associated with developmental complications and increased morbidity and mortality particularly among pre-term infants born before the 32nd week of gestation (1). Following birth, a rapid adaptation of the digestive system is needed to facilitate nutrient uptake and growth, which is of particular importance after pre-term birth where feeding intolerance and maldigestion are common problems (2). The newborn piglet, both pre-term and term, is a clinically relevant animal model for translational studies of newborn human infants (3–5). It has been extensively used to investigate the development of the digestive and the immune system as well as brain development (6–9). On this background, knowledge on maturation of the digestive system, and the identification of diets and feeding strategies that are adapted to and promote maturity of the digestive system, are key to prevent pre-maturity-derived clinical complications.

The intestinal responses to pre-term birth end enteral feeding have been well-characterized, however, less is known about the responses of the accessory digestive organs, such as the pancreas. The pancreas is immature at birth and undergoes post-natal maturation, including a rapid growth within the first 2 days of life with about 80% of mass gain (10) and an increase in exocrine enzymes production and secretion (11). Birth and post-natal enteral nutrition have been shown to stimulate gut maturation (12, 13), specially due to colostrum-borne growth factors that stimulate post-natal pancreatic development (14, 15). Noteworthy specifically for humans, amylase, lipase and esterase enzyme activities are natural components of human breast milk that may play an important role for luminal digestion of dietary lipids in the human neonate in the immediate post-natal period (16, 17).

Previous studies have shown that mature exocrine pancreatic function is not reached before 1 month of age in both pre-mature and term infants (18–20). Moreover, it has been shown that different diets can cause changes in pancreatic enzymes composition and secretion in formula-fed infants (18, 21), indicating that the enzymatic composition of pancreatic secretions can be manipulated (22). In pigs, exocrine pancreatic secretion has been shown to be low from birth to weaning and to increase thereafter (23, 24), together with marked changes in pancreatic enzyme composition around weaning (25, 26). Few studies on development of pancreatic function have investigated the immediate post-natal period.

To study pancreatic adaptation to pre-maturity and dietary supplementation with bovine colostrum, we performed a series of experiments using piglets delivered by cesarean section at either term or pre-term stage, and fed enteral or parenteral diets. Currently, there are ongoing clinical trials on pre-term infants testing bovine colostrum as a first feed (27) or as a fortifier (28). These trials in infants are partly based on studies in pre-term piglets showing improvements of colostrum as a first diet (29), or as a fortifier (30, 31). Therefore, the current study compiles three experiments addressing scientific questions related to the influence of pre-term birth and post-natal responses to diet on the development of pancreatic digestive enzymes. In the first experiment, piglets born either pre-term or term were compared according to either identical post-conceptional or post-natal age, enabling us to discriminate between factors related or unrelated to age at birth. In the second and third experiments, we investigated how dietary supplementation with bovine colostrum, either as the first feed or as a fortifier for pre-term or term piglets, affected the exocrine pancreas. This included observations of both the immediate and the protracted responses, allowing us to investigate whether early responses in the exocrine pancreas are persistent or temporary.

## Materials and Methods

### Pigs, Dietary Interventions, and Experimental Design

The Danish Animal Experiments Inspectorate approved all animal experiments (license no. 2014-15-0201-00418), which is in accordance with the guidelines of Directive 2010/63/EU of the European Parliament and the ARRIVE guidelines (32).

Neonatal piglets were delivered by elective cesarean section from pregnant sows (Danish Landrace x Large White x Duroc) either pre-term (90%) at day 106 of gestation, or term (100%) at day 117 of gestation. Sedation, anesthesia and resuscitation were done as described by Sangild et al. (3), Shen et al. (29), Andersen et al. (33), and Hansen et al. (34). After cesarean delivery, all piglets were immediately transferred to a neonatal piglet intensive care unit, where they were reared in temperature-, moisture- and oxygen-regulated incubators. To provide enteral and parenteral nutrition, all pigs were fitted with an orogastric tube (6 Fr; Pharmaplast, Roskilde, Denmark) and an umbilical arterial catheter (4 Fr; Portex, Kent, UK). To secure passive immunization and prevent infection, piglets were immunized with 12–25 ml/kg of their mother's blood plasma, which was infused via the arterial catheter within 24 h. Piglets were weighed daily and nutritional intake was adjusted according to body weight.

#### Experiment 1: Effect of Post-conceptional vs. Post-natal Age

**Figure 1:**
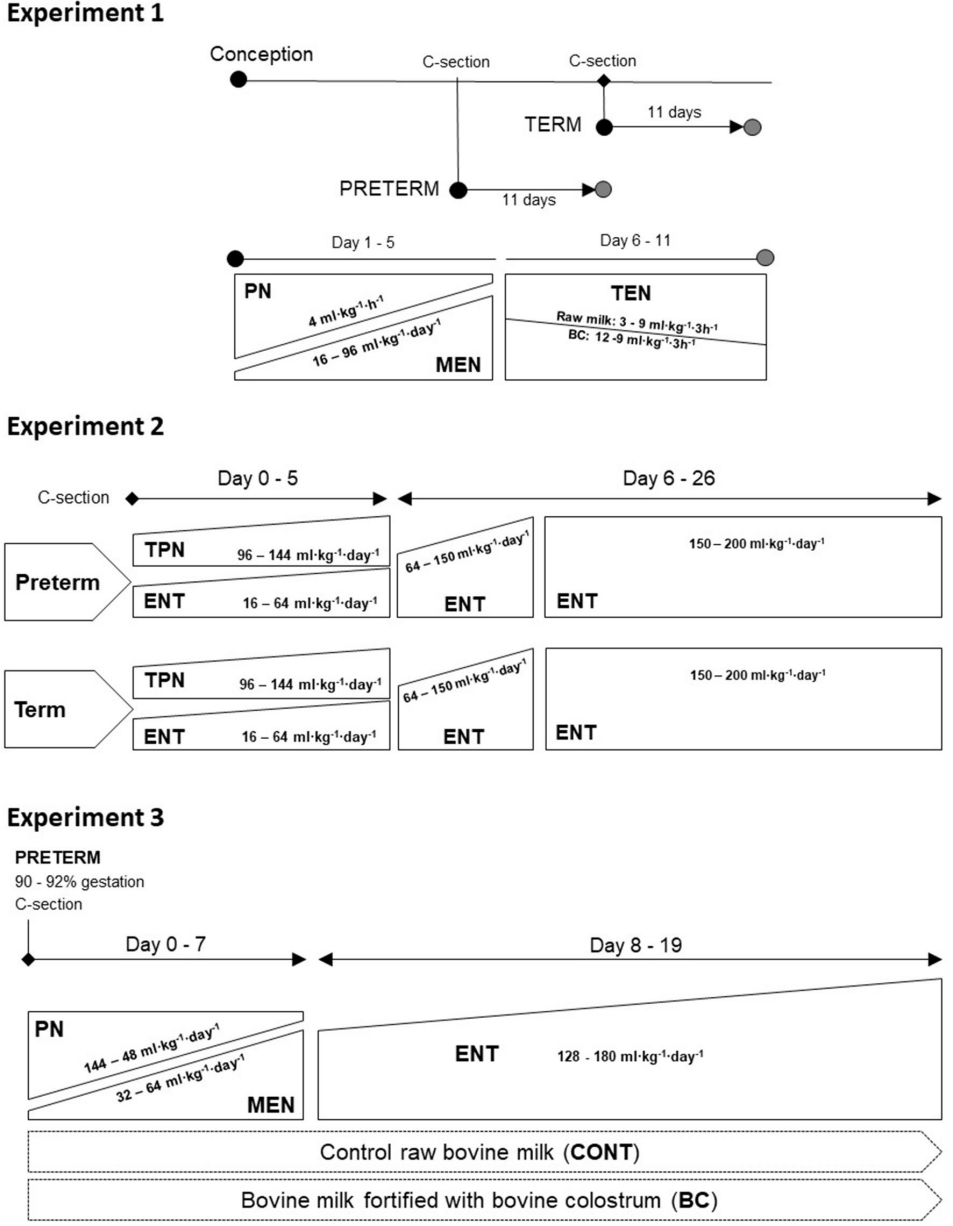
Experimental design of each experiment included in the study. Experiment 1 (top): Cesarean delivered pre-term and term piglets were investigated at birth, i.e., day 1 (TERM-d1 and PRE-TERM-d1, both *n* = 17), and at post-natal day 11 (TERM-d11, *n* = 24 and PRETEM-d11, *n* = 26). Small amounts of enteral nutrition (MEN) were provided together with parenteral nutrition (PN) support for the first 5 days, followed by total enteral nutrition (TEN) until day 11. The enteral nutrition was composed of pure bovine colostrum the first 5 days, while during days 6–11 it was combined with increasing amounts of bovine milk. Experiment 2 (middle): Pre-term and term piglets delivered by cesarean section divided into two groups: total parenteral nutrition (TPN) or parenteral nutrition with minimal enteral nutrition with bovine colostrum (ENT) for the first 5 days. From day six parenteral nutrition was discontinued, and all pigs were given full enteral nutrition in increasing amounts of bovine milk until the end of the experiment, at day 26. Pancreata were sampled on days 1, 5 and 26 from pre-term and term piglets resulting in groups as follows: pre-term 1d (*n* = 10); pre-term 5d ENT (*n* = 9); pre-term 5d TPN (*n* = 12); pre-term 26d ENT (*n* = 16); pre-term 26d TPN (*n* = 16). Term 1d *n* = 11); term 5d ENT (*n* = 11); term 5d TPN (*n* = 10); term 26d ENT (*n* = 11); term 26d TPN (*n* = 10). Experiment 3 (bottom): Pre-term piglets delivered by cesarean section were given parenteral nutrition (PN) for seven days together with increasing amounts of minimal enteral nutrition (MEN) with either colostrum-fortified bovine milk (BC; *n* = 12) or raw bovine milk (CONT; *n* = 10). Total enteral nutrition (ENT) was continued with the same regimen from day 8 until the end of the experiment on day 19.

Piglets were delivered by cesarean section either pre-term on day 106 of gestation (*n* = 43, from 2 sows) or at full term on day 116 of gestation (*n* = 41, from 2 sows). Pancreatic tissue was collected from pre-term and term piglets euthanized either immediately after birth (TERM-d1 and PRE-TERM-d1, both *n* = 17) or after rearing in the intensive care unit until day 11 (TERM-d11, *n* = 24, and PRETEM-d11, *n* = 26), see Figure 1 top.

Pigs received continuous parenteral nutrition (PN) at 96 ml·kg^−1^·day^−1^ via the umbilical catheter. PN was adjusted from commercially available products (Kabiven, Soluvit, and Vitalipid; Fresenius Kabi, Bad Homburg, Germany), as previously presented in detail (35). PN was supplied from birth to post-natal day 5, when catheters were removed. Enteral nutrition (EN) was provided in increasing amounts with bovine colostrum until day 6, together with PN. From day 6 to 11, all pigs were given a diet with decreasing proportions of colostrum and increasing proportions of raw bovine milk as previously detailed (35).

At the end of the experiment, pigs were anesthetized with Zoletil (0.1 ml/kg i.m.), and then, euthanized with an intracardiac injection of 5 ml of 20% pentobarbital. The abdomen was opened and pancreatic samples were quickly collected and snap-frozen in liquid nitrogen and stored until further analysis.

#### Experiment 2: Influence of Early Post-natal Enteral Nutrition Stimulation in Term and Pre-term Piglets

**Figure 2:**
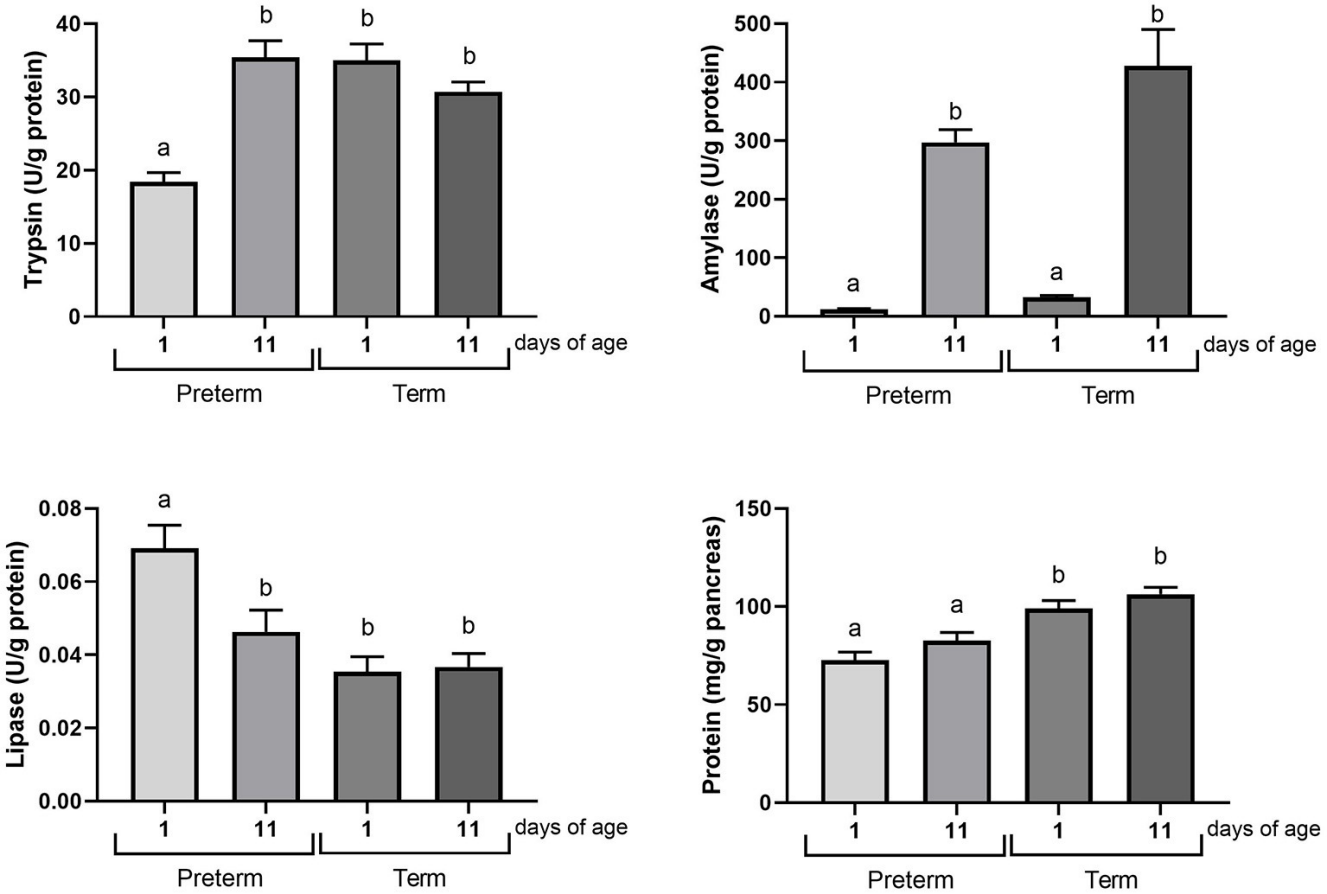
Exocrine pancreatic function (trypsin, amylase and lipase enzymatic activities, and protein content) in cesarean section delivered 11 days pre-mature and term piglets were investigated at birth and at post-natal day 11. Term 1d, *n* = 15; term 11d, *n* = 15; pre-term 1d, *n* = 15, and pre-term 11d, *n* = 15. Data presented as mean ± SEM. Statistical analysis by two-way ANOVA with Tukey's multiple comparisons *post-hoc* test. Different letters indicate statistically significant differences between groups (*p* < 0.05).

Piglets from seven sows were delivered by cesarean section either pre-term on day 106 of gestation (*n* = 63, from four sows) or at full term (*n* = 53, from three sows), as described in Experiment 1. Pre-term and term piglets were provided with either (1) total parenteral nutrition (TPN) with no enteral stimulation of the pancreas via the luminal side, or (2) a combination of parenteral and enteral nutrition to induce luminal stimulation of the pancreas, i.e., referred to as ENT, see Figure 1 middle. Parenteral nutrition was infused via an umbilical artery catheter, as described for Experiment 1. Infusion rates are indicated in Figure 2, and the nutrient composition was (in g/l) carbohydrates (71.4), amino acids (44.6), and fat (30.9) with a total energy density of 745 kcal/l (Kabiven, Vitalipid, Soluvit, and Vamin; Fresenius Kabi, Bad Homburg, Germany) (33, 34). While the TPN group received only parenteral nutrition, the ENT group was nurtured with a combination of parenteral nutrition and enteral nutrition provided as blouses of bovine colostrum (170 g/l colostrum powder; Biofiber Damino, Vejen, Denmark) every 3 h at increasing amounts 16–64 ml·kg^−1^·day^−1^. On day 5, parenteral nutrition was discontinued for all pigs and umbilical catheters were removed. Afterwards, pigs were all given bovine milk (64–150 ml·kg^−1^·day^−1^) until day 9 and were subsequently transitioned to reconstituted whole milk powder (150–200 ml·kg^−1^·day^−1^) until day 26 (Arla Foods Ingredients, Viby, Denmark). The orogastric feeding tubes were removed on day 8 and pigs were trained to voluntary drinking from a trough thereafter. The dosing strategies for both TPN and enteral feeding were adjusted to be comparable with those used for moderately pre-term infants (36–40). Of the total of 116 piglets, subgroups of pre-term and term pigs were euthanized and pancreatic tissue were collected on days 1, 5, and 26, resulting in ten experimental groups: pre-term 1d (*n* = 10); pre-term 5d ENT (*n* = 9); pre-term 5d TPN (*n* = 12); pre-term 26d ENT (*n* = 16); pre-term 26d TPN (*n* = 16). Term 1d (*n* = 11); term 5d ENT (*n* = 11); term 5d TPN (*n* = 10); term 26d ENT (*n* = 11); term 26d TPN (*n* = 10). Details on housing conditions and antibiotics treatment are presented in Andersen et al. (33) and Hansen et al. (34).

#### Experiment 3: Bovine Colostrum Fortification vs. Raw Bovine Milk in Pre-term Piglets

**Figure 3:**
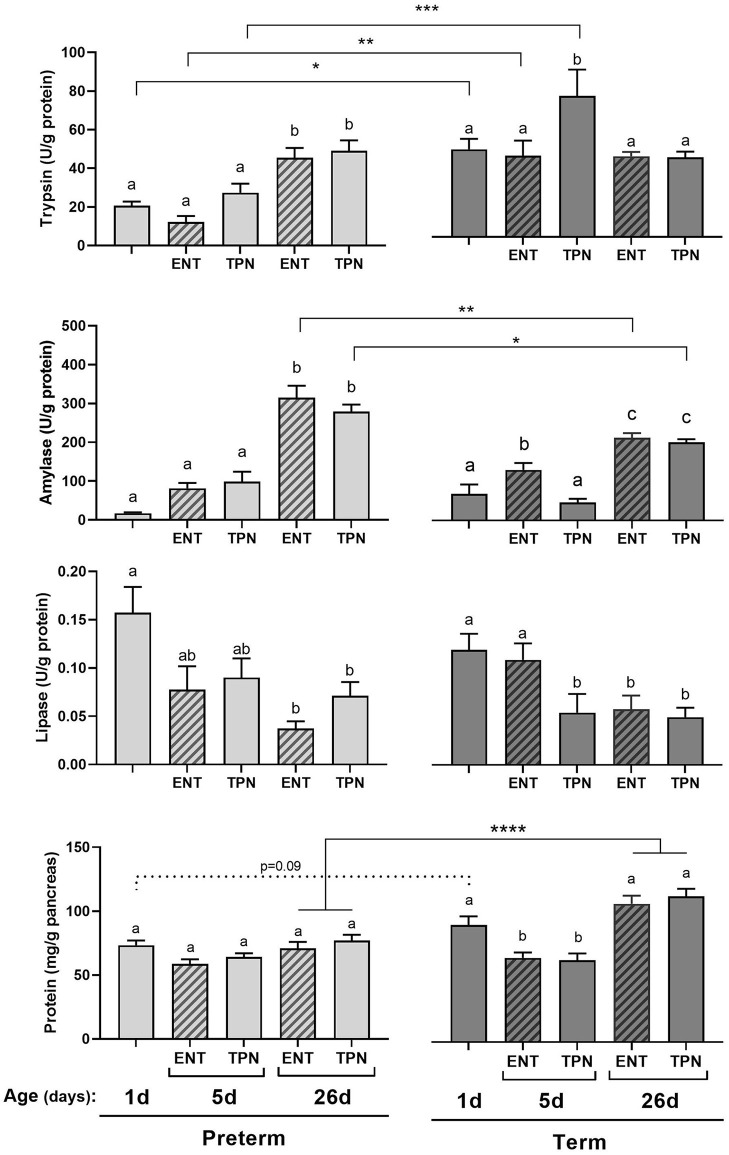
Exocrine pancreatic function (trypsin, amylase and lipase enzymatic activities and protein content) in pre-term or term piglets delivered by cesarean section divided into two groups: total parenteral nutrition (TPN) or parenteral nutrition with minimal enteral nutrition with bovine colostrum (ENT) for the first five days. From day 6 all groups ceased parenteral nutrition and were given enteral nutrition in increasing amounts of bovine milk until the end of the experiment, at day 26. Pre-term 0 days, *n* = 10; pre-term 5d ENT, *n* = 9; pre-term 5d TPN, *n* = 12; pre-term 26d ENT, *n* = 16; pre-term 26d TPN, *n* = 16; Term 0 days, *n* = 11; term 5d ENT, *n* = 11; term 5d TPN, *n* = 10; term 26d ENT, *n* = 11; term 26d TPN, *n* = 10. Data presented as mean ± SEM. Statistical analysis by two-way ANOVA with Tukey's multiple comparisons *post-hoc* test and pair-wise comparisons. Different letters indicate statistically significant differences between groups of the same gestational age at birth. Statistical difference in pair-wise comparisons between corresponding groups of different gestational ages indicated with *p < 0.05, **p < 0.01, ***p < 0.001, and ****p < 0.0001.

Forty-four piglets from two sows were delivered by cesarean section pre-term and distributed in two experimental groups (Figure 1 bottom), as described earlier (41). Pigs were fed an enteral diet based on diluted (66% v/v) bovine milk (CONT, *n* = 10) or diluted milk fortified with bovine colostrum powder at 59 g/l (Biofiber Damino) (BC, *n* = 12). Diets were provided in increasing amounts from 32 to 64 ml·kg^−1^·day^−1^. All pigs received parenteral nutrition support until day 8, after which they were transitioned to voluntary drinking from troughs with increasing amounts, from 128 to 180 ml·kg^−1^·day^−1^. Orogastric tubes were removed on day 12. From day 5 to 19, minerals and vitamins were added to both diets (Pediatric Seravit, 15 g/l, Nutricia and Revolyte Nutrition). On day 19, piglets were anesthetized and euthanized, pancreatic tissue samples were collected and snap-frozen for later analysis of enzymatic activity, as previously described. For details on feeding volumes and dietary composition see Figure 3 and Ahnfeldt et al. (41).

Antibiotics were administered prophylactically twice daily on days 8, 9 and 10 intra-gastrically with a combination of Amoxicillin/clavulanic acid (“2care4”; 50 mg amoxicillin/kg), Gentamicin (Gentocin Vet.; 2.5 mg/kg) and Metronidazole (Flagyl; 10 mg/kg).

### Pancreatic Enzymology

Pancreata were homogenized 1:10 (w/v) with ice-cold 0.2M Tris-HCl buffer + 0.05M CaCl_2_, pH 7.8, with a glass homogenizer, centrifuged at 24,000 x g for 30 min at +4°C, and the supernatants were analyzed. The protein concentration was determined by the Lowry method with a modification for 96-well microplates (42) and using purified BSA as the standard. After activation with enteropeptidase, trypsin activity was determined spectrophotometrically using a microplate modification (23) of the method of Fritz et al. (43), with benzoyl-DL-arginine-4-nitroanilide (BAPNA) as the substrate. Amylase activity was analyzed with a detection kit according to the manufacturer's instructions (Infinity Amylase Liquid Stable Reagent; Thermo Fisher Scientific Inc., USA) modified for microplate usage. Lipase activity was measured using a long-chain substrate by the Lipase Detection Kit (Colorimetric) (ab102524, Abcam) according to the manufacturer's instructions. Spectrophotometric measurements of absorbance were done in a 96 well plate reader SpectraMax i3x (Molecular Devices). The enzyme activities were expressed relative to protein concentration, as U/g protein.

### Calculations and Statistics

All data are presented as mean ± standard error of the mean (SEM). Statistical analyses were done using SAS 9.4 (SAS Inc, USA) and graphs were done in Prism v9 (GraphPad Software, USA, www.graphpad.com). Experiment 1 was analyzed by two-way-ANOVA, with gestational age and post-natal age as main factors, and Tukey's multiple comparisons *post-hoc* test. Experiment 2 was analyzed by two-way ANOVA, with post-natal age and treatment as main factors, with Tukey's multiple comparison within each post-conceptional age at birth (pre-term or term), indicated with different letters; and Sidak's multiple comparison test was used between the corresponding age and treatment group from the different post-conceptional age at birth, indicated with symbols. Experiment 3 statistical analysis was done by Kruskal-Wallis non-parametric unpaired *t*-test. Differences were considered significant when *p*-value < 0.05. Statistical differences are indicated with different letters or symbols [*p* < 0.01 (^**^), *p* < 0.001 (^***^), *p* < 0.0001 (^****^)]. Tendencies were considered when 0.05 < *p*-value < 0.1.

## Results

### Experiment 1

We investigated the influence of post-conceptional age and post-natal age on the development of the exocrine pancreatic function, by comparing piglets born 11 days pre-term to piglets born term, and as both groups were evaluated also at post-natal day 11, it allows us to compare pigs of identical post-conceptional age (i.e., PRE-TERM-d11 vs. TERM-d1) age, and identical post-natal age (i.e., PRE-TERM-d11 vs. TERM-d11) (Figure 2).

While pancreatic trypsin activity was lower in pre-term piglets at birth (*p* < 0.05) compared to all other groups, we found the opposite for lipase activity, which was higher in pre-term piglets at birth (*p* < 0.05) compared to all other groups. Accordingly, pre-term pigs at post-natal age of 11 days reached the same plateau for trypsin and lipase as was seen for term pigs at both day 1 and day 11. This indicates that development of trypsin and lipase activities is more under the influence of post-conceptional (fetal) age than post-natal age. In contrast, amylase activity showed a clear dependency of post-natal age rather than post-conceptional age, as it was >30 fold higher in both pre-term and term piglets at post-natal day 11 compared to the levels at birth (day 1) (*p* < 0.0001).

We also determined the protein content in the pancreatic homogenates, comprising the water-soluble proteins primarily consisting of digestive enzymes. Thus, the protein measured in the pancreatic samples was considered to show the total amount of exocrine enzymes, and in general it was higher in term compared with pre-term pigs at both 1d and 11d (*p* < 0.05). This indicates that the total enzyme content in pancreatic tissue is under dual influence of both post-conceptional age, as indicated by higher levels in term vs. pre-term pigs on both day 1 and 11, and post-natal age, as indicated by higher level in term vs. pre-term at day 11. This is in line with the observation that lipase, trypsin and amylase showed distinct developmental trajectories relative to post-conceptional and post-natal age.

### Experiment 2

To investigate the influence of early enteral nutrition we compared piglets, born both pre-term and term, given either a combination of enteral and parenteral nutrition (ENT) or parenteral nutrition only (TPN) for the first 5 days after birth. To determine whether this 5-day treatment had short or long-term effects on pancreas development, results are presented for each treatment at day 1, 5 and 26 (Figure 3). Hence, piglets were compared according to gestational age at birth (i.e., pre-term *vs*. term), to post-natal age (i.e., 1, 5 or 26 days old), and by feeding strategy early in life (i.e., TPN *vs*. ENT).

We found that pancreatic trypsin activity was low in pre-term piglets at birth but it had increased to levels similar to that of newborn term pigs by day 26 (Figure 3, top graph). The different dietary treatments (ENT and TPN) during the first 5 days did not influence trypsin activity in pre-term piglets, while term piglets on TPN showed the highest level of trypsin. Any differences in trypsin activity levels due to dietary treatments did not persist after 26 days.

Amylase activity was low at birth and increased with age for both pre-term and term pigs (Figure 3, middle-top graph). This was largely independent of dietary treatment (TPN or ENT) in the pre-term pigs but term ENT piglets of 5 days of age showed a substantial increase in amylase activity relative to TPN. Like for trypsin, this indicates responsiveness to enteral nutrition among term pigs, whereas pre-term pigs are not responsive. Moreover, pancreatic amylase activity reached a higher level in 26 days old pre-term piglets compared with term piglets this difference was mainly caused by higher protein concentration in term pigs (Figure 3, bottom graph).

Pancreatic lipase activity was high at birth, for both pre-term and term pigs, and showed a trend to decrease until reaching the lowest levels at either 5 or 26 days of age (Figure 3, middle-bottom graph). Noteworthy, term pigs given ENT did show numerically higher levels of lipase activity relative to TPN pigs on day 5. Although this did not reach statistical significance, it is in line with the observations from trypsin and amylase, that term piglets are responsive to stimulation from enteral nutrition, while pre-term pigs are not.

Finally, we found that the pancreatic protein content was at similar levels in pre-term piglets across all ages and treatments (Figure 3, bottom graph). In contrast, the protein levels had decreased after day 5 in term piglets, after which it increased markedly (~+50%) until 26 days of age. This was not influenced by dietary treatment at neither 5 nor 26 days of age.

### Experiment 3

**Figure 4:**
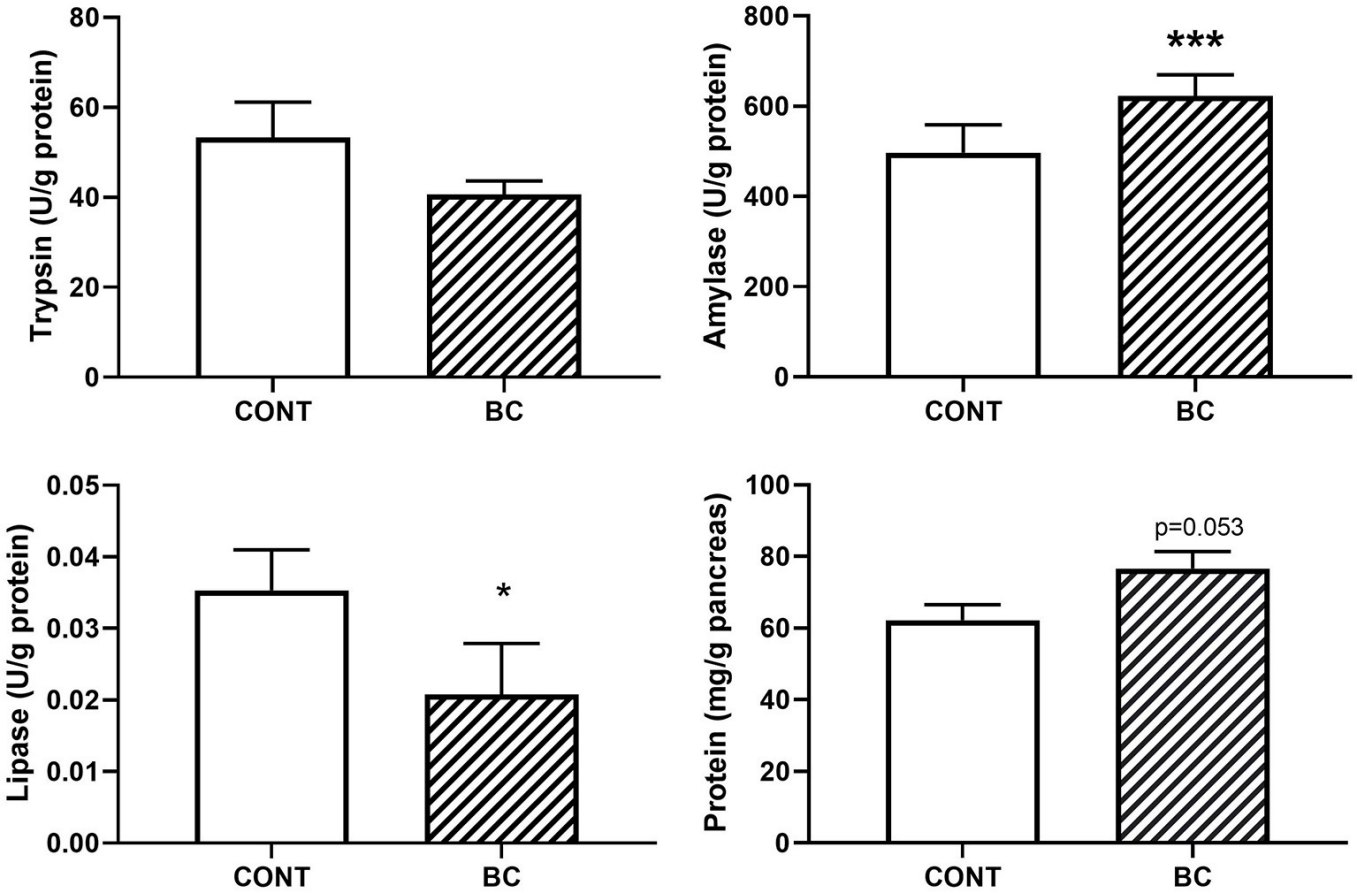
Exocrine pancreatic function (trypsin, amylase and lipase enzymatic activities and protein content) in pre-term piglets delivered by cesarean section given parenteral nutrition for seven days together with increasing amounts of minimal enteral nutrition with either colostrum-fortified bovine milk (BC) or raw bovine milk (CONT). Total enteral nutrition was continued with the same regimen from day 8 until the end of the experiment on day 19. CONT, *n* = 10; BC, *n* = 12. Data presented as mean ± SEM. Statistical analysis done by Kruskal-Wallis non-parametric unpaired *t*-test. Statistical difference between groups are indicated with *p < 0.05 and ***p < 0.001.

While in experiment 2 we show the influence of enteral nutrition (ENT) vs. parenteral nutrition (TPN), the aim of experiment 3 was to investigate the influence of colostrum fortification in the pre-mature pigs during a longer post-natal period. We compared a group of pre-term piglets fed with diluted milk, which was predicted to give restricted growth (referred to as control group, CONT), with pre-term piglets fed with the same diluted milk fortified with bovine colostrum, predicted to give normal growth (referred to as the colostrum group, BC). Thus, this study focused on the effects of dietary supplementation with bovine colostrum on pancreatic development of pre-term piglets after 19 days (Figure 4).

A reduction in lipase and trypsin (only numerically) activity was found in BC fed pre-term pigs relative to the control group. However, this was partly a result of higher pancreatic protein content in the BC group. In contrast, amylase activity was higher in the colostrum group confirming the notion from experiments 1 and 2, that these three enzymes display distinct developmental trajectories relative to age, enteral nutrition and type of diet.

## Discussion

Development of the porcine exocrine pancreas during pregnancy and post-natal life has been studied by Sangild et al. (44). Using pig fetuses, they found that amylase and trypsin activities per mg of protein increased toward term, and that this increase was positively correlated with plasma cortisol. They also showed that fetal development of the exocrine pancreas could be accelerated when the fetuses were infused with cortisol, indicating the presence and responsiveness of cortisol-receptors even before birth. Hence, this has clinical implications in the situation where pre-term delivery is anticipated, as cortisol treatment may improve pancreas exocrine function and support early life digestive function for vulnerable pre-term neonates. Even though the porcine placenta is less permeable to cortisol compared to human placenta, the shown responsiveness to cortisol in porcine fetuses under experimental conditions adds valuable information that may apply to human fetuses. Others have tried to compare the ontogeny of pancreatic function in newborn pigs and humans (20, 22). In a study from Zoppi et al. duodenal catheters were inserted to collect pancreatic juice from pre-term and term babies upon stimulation with pancreozymin and secretin (18). Partly in line with our observations, they found that pre-term neonates had lower levels of amylase, trypsin and lipase relative to term infants. They also showed that feeding a milk formula containing starch increased amylase activity in particular, whereas lipase activity was independent from the dietary fat content. This suggests a discrepancy between the different pancreatic enzymes with regard to their dependency on substrate in the diet. Even though the work of Zoppi et al. represented secreted pancreatic enzymatic activity (18), our observations are in line with regard to amylase activity in pancreatic tissue, which developed rapidly after birth in both pre-term and term pigs, whereas lipase activity was largely unaffected. Although we do see a reduction in lipase activity relative to protein with increasing age, it should be noted that this results mainly from the increasing pancreatic total protein content rather than a decrease in lipase activity.

Although there are some studies that have assessed pancreatic function via analysis of pancreatic secretions, such as duodenal aspirates (18, 45), there is a paucity of data on pancreatic development following pre-term delivery and the responsiveness to post-natal dietary regimens, like parenteral and enteral nutrition. As the adaptation mechanisms particularly following pre-term birth are largely unexplored, we have performed a series of animal experiments to elucidate the effects of post-conceptional (fetal) age, post-natal age, enteral feeding and colostrum fortification, on the development of the exocrine pancreatic enzymes in young piglets.

### Effects of Age on Exocrine Pancreatic Enzymes: Post-conceptional vs. Post-natal Age

We investigated pancreatic enzyme activities in pigs of either the same post-conceptional age or the same post-natal age. Our results showed that trypsin activity increases markedly during the late fetal period, i.e., the last 11 days of a full gestation, to reach a plateau, which remains constant after term birth. In contrast, trypsin activity following pre-term birth continued to increase after birth that by day 11 had reach similar levels to those of term pigs on day 1, and with intermediate values in 5 day-old pre-term piglets. Collectively, these observations indicate that trypsin development was more determined by post-conceptional age than by post-natal age, and that pancreatic trypsin develops much during the late fetal period. Our finding that trypsin activity has reached a plateau at the time of birth is partly in line with studies in term human infants, in which pancreatic trypsin activity at birth was estimated to be 90% of the levels in childhood (19) and increased within the first 3 post-natal weeks (46).

In contrast to trypsin, post-natal age rather than post-conceptional age was the main determining factor for pancreatic amylase development. On post-natal day 11, the levels of amylase activity were profoundly higher in both pre-term and term piglets compared with their levels at birth, while the levels were largely similar for pigs of the same post-natal age, i.e., term day 1 vs. pre-term day 1 and term day 11 vs. pre-term day 11. Others have shown that pancreatic amylase activity is very low until birth and that it increases to adult levels within the first 3 post-natal months (47). In newborn term piglets, pancreatic amylase has been shown to appear during the last 2 weeks of fetal life even though it increases from post-natal day 0 to day 3 (44). In accordance with this, we found higher amylase levels at birth in term vs. pre-term piglets, but this difference was minor if compared with the levels at 11 days after birth. Consequently, from our results we can conclude that development of amylase activity is primarily driven by post-natal factors, further discussed under the section on dietary effects on exocrine pancreas development.

We found that pancreatic lipase levels were highest at birth, especially in pre-term pigs, with a decreasing trend in the post-natal period for both pre-term and term pigs, which was inverse to the development of trypsin. However, since we present our enzyme activity results normalized to the total protein concentration and there is an increase in pancreatic protein content in term pigs, taken together, it indicates that lipase activity expressed per gram of tissue is largely constant. Although a limitation in our study is the lack of information on daily volume of pancreatic secretions, others have shown that pancreatic lipase activity increases during the first 10 post-natal weeks (46) and that weaning appears to stimulate this increase (48). Likewise, duodenal aspirates from human infants indicated a very low level of lipase activity at birth relative to the level at >2 years of age (19). In the present study, the commercial method used for lipase activity quantification was based on a long-chain fatty acid substrate that possibly has an impact on the specificity for different pancreatic lipases, e.g., pancreatic lipase and bile-salt stimulated lipase (carboxyl ester lipase). The former being the dominant lipase in the weaned/adult while the latter could be a more important lipase in the young pig on a milk diet consisting of medium-chain fatty acids (48).

### Effects of First Feeding on Exocrine Pancreatic Enzymes–TPN vs. ENT

Pre-term birth is often followed by complications such as breastmilk unavailability and/or an insufficient suckling reflex of the pre-term infant. Therefore, enteral tube feeding with donor milk or commercial milk formulas are common clinical practices in neonatal intensive care units. Moreover, pre-term infants who particularly cannot tolerate full enteral feeding usually require some level of support with parenteral nutrition. Even though the support with parenteral nutrition is essential for survival during the immediate post-natal period, it does not provide any enteral stimuli of the digestive tract or its accessory organ the exocrine pancreas, which have been proven to be of relevance. Nutrients are provided parenterally *in utero*, but pig fetuses also swallow substantial amounts of amniotic fluid that provide important enteral stimulation (49), nonetheless, at birth the newborn must abruptly adapt to full enteral nutrition (50). Previous studies showed that piglets, both pre-term and term, fed TPN for 6 days after birth had lower pancreatic weight (4), and thus, showing that the first enteral feeding provides important trophic effects (51).

Interestingly, we found a discrepancy in enzyme activities for the three studied enzymes with regard to the route of nutrition, i.e., enteral (ENT) vs. parenteral (TPN) on day 5 after birth. While the enzyme activities in pre-term piglets were not influenced by route of nutrition, clear effects were observed in term piglets, in the sense that trypsin activity was increased, and amylase and lipase were decreased in 5 day old TPN-fed term pigs. We speculate that the increase in trypsin may be explained by the absence of enteral stimulation of pancreatic secretion causing accumulation of the enzyme in the tissue. This TPN-induced complication may be further exacerbated by the rapid post-natal increase in pancreatic weight and the volume of pancreatic secretions (52, 53). Moreover, trypsin activity seemed to be the least sensitive to the diet among the three measured enzymes, regulated by the stimulation or lack thereof of pancreatic secretion.

Conversely, pancreatic amylase activity decreased due to TPN nutrition in term piglets at 5 days of age indicating that the development of amylase is dependent on enteral stimulation *per se* as well as the nature of the enteral diet since colostrum induced higher amylase levels than regular milk. The difference between trypsin and amylase enzymes was also observed in previous studies that showed decreased pancreatic trypsin activity while amylase activity was increased in both pre-term and term piglets given enteral nutrition for 6 days after birth compared to piglets given TPN (4).

Likewise, lipase activity was decreased in TPN-nurtured 5 days old piglets in the same direction as older piglets, indicating that absence of enteral stimulation caused a reduced lipase activity in the pancreas. Very low birth weight infants fed fortified human milk or formulas, mostly differing in fatty acid composition, showed decreased lipase activity by formula feeding and a non-significant increase in trypsin activity in fortified human milk (54). Thus, the post-natal development of pancreatic lipase activity seems to depend on dietary fat content, which would also agree with the need for higher levels of lipase activity at birth, due to the higher fat content in colostrum, and the decrease in lipase activity during the suckling period, when carbohydrates become the main dietary component. This maturational effect on pancreatic lipase can also be observed in pre-term piglets fed with colostrum-fortified milk in experiment 3.

Pancreatic development starts *in utero* with exocrine enzymes increasing with fetal age (26). At birth, there is a decrease in pancreatic enzymatic activities due to the stimulating effect of colostrum intake in pancreatic secretion (53). Term born piglets undergo a gradual increase in the synthesis and secretion of pancreatic enzymes/zymogens during the suckling period (0–5 weeks) (24–26, 48). Indeed, at 26 days after birth no differences were found in the enzymes due to either parenteral of enteral feeding at day 5 after birth between the pre-term of term piglets, indicating that the initial effects of enteral vs. parenteral feeding, did not have persistent long-term effects on the exocrine pancreatic function.

### Effects of Dietary Fortification With Colostrum on Exocrine Pancreatic Enzymes

Pancreatic enzymes are required to support growth via digestion and absorption of nutrients, as well as supplying trophic effects on the intestinal tissue (51, 53). Moreover, pancreatic enzymes also have a protective effect on the digestive system due to antimicrobial activity of pancreatic secretions (55). We found that bovine colostrum-fortification supported pancreatic function in pre-term piglets as shown by the increased pancreatic total protein content and amylase activity, while lipase and trypsin were reduced (albeit only numerically for trypsin). From the findings in experiment 1 and 2, we interpret that the effects of colostrum particularly on protein and amylase, is indicatory of an improved maturation of the exocrine pancreas. Although at this stage the exact mechanisms are elusive, we hypothesize that they relate to the content of bioactive molecules, such as hormones and enzymes, in colostrum that stimulate growth and maturation in the digestive tract (56). Although further research is required to understand the exact mechanisms by which colostrum stimulates the exocrine pancreas, the current data may have important perspectives for formulation of milk-diets for vulnerable newborn infants.

## Conclusions

Collectively, the pancreatic digestive enzymes showed asymmetric changes during the post-natal period for both pre-term and term neonates, and showed discrepancies in the responsiveness to enteral feeding. Development of trypsin activity was more determined by post-conceptional (fetal) than post-natal age, while lipase and in particular amylase were more affected by post-natal age, and showed a dependency on the type of enteral nutrition (milk vs. colostrum).

## Data Availability Statement

The raw data supporting the conclusions of this article will be made available by the authors, without undue reservation.

## Ethics Statement

The animal study was reviewed and approved by Danish National Committee of Animal Experimentation (license no. 2014-15-0201-00418).

## Author Contributions

PS and TT: planned the research. EA and KP: data analysis. EA, BW, and TT: interpretation of results and wrote the manuscript. All authors read and approved the final paper.

## Conflict of Interest

The authors declare that the research was conducted in the absence of any commercial or financial relationships that could be construed as a potential conflict of interest.
